# The Work Softening Behavior of Pure Mg Wire during Cold Drawing

**DOI:** 10.3390/ma11040602

**Published:** 2018-04-13

**Authors:** Liuxia Sun, Jing Bai, Feng Xue, Chenglin Chu, Jiao Meng

**Affiliations:** 1 School of Materials Science and Engineering, Southeast University, Jiangning, Nanjing 211189, China; spring3616@126.com (L.S.); xuefeng@seu.edu.cn (F.X.); clchu@seu.edu.cn (C.C.); joejoe1st@yeah.net (J.M.); 2 Jiangsu Key Laboratory for Advanced Metallic Materials, Jiangning, Nanjing 211189, China

**Keywords:** pure Mg, cold drawing, microstructure, texture, work softening

## Abstract

We performed multiple-pass cold drawing for pure Mg wire which showed excellent formability (~138% accumulative true strain) at room temperature. Different from the continuous work hardening occurring during cold drawing of Mg alloy wires, for pure Mg, an initially rapid increase in hardness and strength was followed by significant work softening and finally reached a steady-state level, approximately 40~45 HV. The work softening can be attributed to the dynamic recovery and recrystallization of pure Mg at room temperature. Meanwhile, an abrupt change in texture component also was detected with the transition from work hardening to softening in the strain range of 28~34%. During the whole drawing, the strongest texture component gradually transformed from as-extruded basal to <10$\bar{1}$0> fiber (~28% accumulative true strain), and then rapidly returned to the weak basal texture.

## 1. Introduction

The deformation behavior of Mg has been studied for several decades but an increasing interest has developed recently because of the potential for using Mg as a biomaterial [1,2,3,4]. Being hexagonal close-packed (hcp) in structures with a c/a value of 1.624 and with non-availability of adequate numbers of independent slip systems, Mg alloys intrinsically have relatively low ductility and poor formability at room temperature. Nevertheless, Mg and its alloys exhibit an excellent formability during cold drawing at room temperature [5,6,7], achieving a large accumulated strain.

The strength and hardness of nearly all alloys generally increase by cold drawing. For example, hardness of cold-drawn metalsis improved up to ~60% for Mg-2Zn [7], ~54% for AZ31 [8] and also for a range of other metals strong hardening has been evidenced [9,10]. Some studies suggested that the hardening behavior is mainly due to the strain-induced accumulation of dislocations and/or twins [11,12]. For most metals, the increase in dislocation density and refinement of microstructure cause the hardness to increase with increasing strain, eventually reaching a saturated value. However, several papers reported that the metals with low melting temperatures such as Al [13], Pb [14], Sn [14], In [14], and Zn [15,16] can exhibit severe plastic deformation induced softening even below the hardness levels after high-temperature annealing [13]. For Mg, a relatively limited high pressure torsion (HPT) deformation leads to a steep increase of hardness and further HPT does not remarkably increase Mg hardness further [17].

Work softening behavior is one of the important characteristics in the cold plastic deformation process of materials at room temperature. The unusual softening in above mentioned low-melting-temperature metals is attributed to high mobility of dislocations and the role of grain boundaries as dislocation sinks [14]. In addition, the different dependency of hardness of Mg on the strain during cold drawing may be caused by its HCP structure, which provides only two independent slip systems (basal slip). According to the Taylor’s model, twins and/or non-basal slips may be activated [18]. This may affect the texture of Mg, which has an important influence on the hardness of Mg during cold deformation [7,19]. So far, the effects of microstructure and texture on work softening behaviors of pure Mg wires by cold drawing are rarely reported [20]. 

In this study, the microstructure, texture and microhardness of cold-drawn pure Mg wires with different accumulative true strain (ATS) were investigated. It is crucial to understand the deformation mechanism of pure Mg during wire cold drawing and clarify the reason behind the different hardness evolution between Mg and its alloys processed by cold drawing, thus providing a guide to produce Mg thin wire for medical or similar applications.

## 2. Materials and Methods 

### 2.1. Materials

The materials employed in this study were pure Mg (>99.9%) billets. They were hot extruded at 650 K to a reduction ratio of ~20. Before extrusion, the billets were subject to a standard pre-heat of 10 min at the extrusion temperature. After extrusion, the pure Mg wire with a thicker initial diameter of 3.0 mm was obtained. Then, as-extruded pure Mg wire was subjected to successive cold-drawing passes of true strain ~7% (except the first two passes ~3%) each pass, until ~138% ATS where fracture occurred frequently (>50% in this work).

### 2.2. Characterization

The microstructures were characterized by an Olympus BHM optical microscope (OM, Olympus, Tokyo, Japan) and a Tecnai G2 transmission electron microscope (TEM, FEI, Eindhoven, the Netherlands) operating at 200 kV. Samples for OM analysis were prepared by conventional mechanically polishing and then etching with acetic picral (0.84 g picric acid, 2 mL acetic acid, 14 mL ethanol, 7 mL H_2_O) for 3~5 s. To obtain TEM image, the specimens were thinned by twin-jet electro polishing in a solution of 5 mL perchloric acid and 95 mL ethanol after punching 3 mm diameter disks. 

The misorientation angle distributions of pure Mg wire with lower strain was observed and analyzed using electron backscatter diffraction (EBSD) within a scanning electron microscope (SEM, FEI, Eindhoven, the Netherlands) with a field-emission gun operated at 20 kV. The specimens for EBSD investigation were mechanically ground with a series of SiC sand papers (400#, 800#, 1200#, 2000#, 3000#, 5000#) and diamond polishing, followed by electrochemical polishing in a solution of 5% nital acid in ethanol at 15~20 V for approx. 30 s. Then, the samples were quickly rinsed with ethanol and dried under a blast of air. 

In addition, the texture was tested by X’pert-PRD X-ray diffractometer (XRD, PANalytical B.V, Almelo, The Netherlands). Samples were prepared by ranging several wires to forming the dimension of 20 mm × 20 mm in the same direction, and then, the samples were carefully ground into a plane. The principal directions of experimental samples are denoted by the extrusion or drawing direction (ED or DD), the transverse direction (TD) and the normal direction (ND). Texture examinations were conducted on the longitudinal sections of wires aligned along the drawing direction using XRD in the back-reflection mode operated at 35 kV. Five incomplete pole figures, i.e., {0002}, {10$\bar{1}$0}, {10$\bar{1}$1}, {10$\bar{1}$2} and {11$\bar{2}$0}, were measured. Then, the orientation distribution functions (ODFs) were calculated from the measured pole figures after background and defocusing correction. The complete pole figures and inverse pole figures based on the corrected ODFs were plotted. The tensile tests were carried out by CMT5105 electronic universal testing machine (Sans, Shenzhen, China) with the tensile axis parallel to the extrusion direction, and the micro-hardness measurement was carried out by FM-700 micro-hardness tester (Future-Tech, Kawasaki, Japan).

## 3. Results

### 3.1. Mechanical Properties

**Figure 1:**
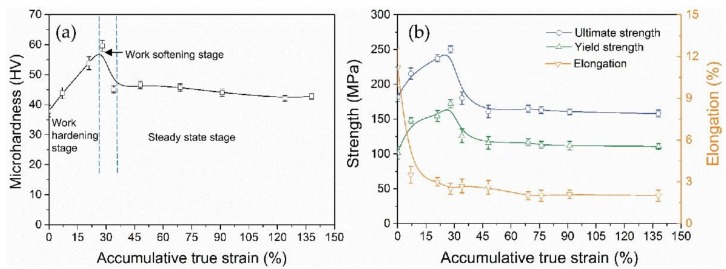
Mechanical properties curves of pure Mg wires, showing the effect of accumulative true strain (ATS) on (**a**) the microhardness, (**b**) the tensile strength and elongation.

The microhardness (HV) curve of pure Mg wires, as a function of ATS, is drawn in Figure 1a, where the HV value initially increases with increasing strain, showing a typical work hardening. After reaching the maximum value at ~28% ATS, the microhardness dramatically decreases to ~45 HV at ~34% ATS and presents an apparent work softening. Subsequently, the curve enters into a steady-state stage with the range of HV values from 40 to 45 until ~138% ATS. A similar trend is also seen from the tensile testing results in Figure 1b, where ultimate strength and yield strength sharply change with the increment of strain, and subsequently reach a plateau. In contrast to the ultimate strength and yield strength, the evolution of elongation is quite different. Elongation rapidly declines with initial strain imposed. Up to ~138% ATS, the elongation remains ~2.5%. To better understand the changes in the microhardness of pure Mg, microstructural observations and texture measurements were performed for as-extruded wires and as-drawn wires.

### 3.2. Microstructure

**Figure 2:**
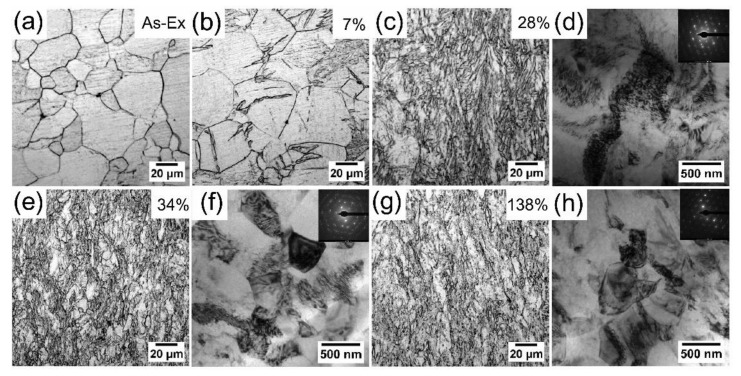
Metallographic photos of (**a**) as-extruded and as-drawn Mg with ATS of (**b**) 7%, (**c**) 28%, (**e**) 34%, (**g**) 138%, and (**d**) (**f**) (**h**) TEM micrograph of wire with corresponding ATS (28%, 34%, 138%).

The microstructures of as-extruded and as-drawn Mg wires are shown in Figure 2. The original microstructure of as-extruded Mg contains an inhomogeneous grain distribution. The grain size ranges from 5 to 60 μm and the average grain size is ~23 μm (Figure 2a). In the incipient stage of drawing (Figure 2b), there are gradually twins appearing in the intragranular zones. As ATS increases, the grains are elongated and the microstructural evolution can barely be distinguished from metallography. However, there are remarkable changes for the substructures observed by TEM. At ATS ~28% (Figure 2d), the dislocation density significantly increases and dislocation cells are formed. The observation is supported by representative selected area diffraction pattern (SADP) inserted in Figure 2d. The diffraction spots are elongated, which confirms the presence of dislocation cells. At ATS ~34% (Figure 2f), dislocation cells gradually transform into smaller subgrains with size of ~450 nm. Simultaneously, some high-angle grain boundaries have formed. This is also confirmed by corresponding SADP, where the elongated diffraction spots locate around near circles, indicating the presence of a large fraction of very small subgrains. These results reveal the occurrence of dynamic recovery and recrystallization (DRX) during cold drawing of pure Mg. At larger ATS ~138% (Figure 2h), dislocation density significantly reduces and more high-angle equiaxed grains appear with size of ~600 nm. From TEM micrograph and SADP, these grains have obviously coarsened in comparison with those presented in Figure 2f. It also can be seen that within the grain interiors the contrast is non-uniform and appear to be complex, thereby implying the presence of a high level of internal stresses. Simultaneously, a carefully inspection of microstructures reveals the grain boundaries are curved and wavy under this experimental condition. These irregular boundaries give evidence indicating the boundaries introduced by cold-drawing are in a non-equilibrium configuration with high energy. These microstructural features are distinctly different from that of cold-drawn Mg alloys with high density dislocations [7].

### 3.3. Texture

**Figure 3:**
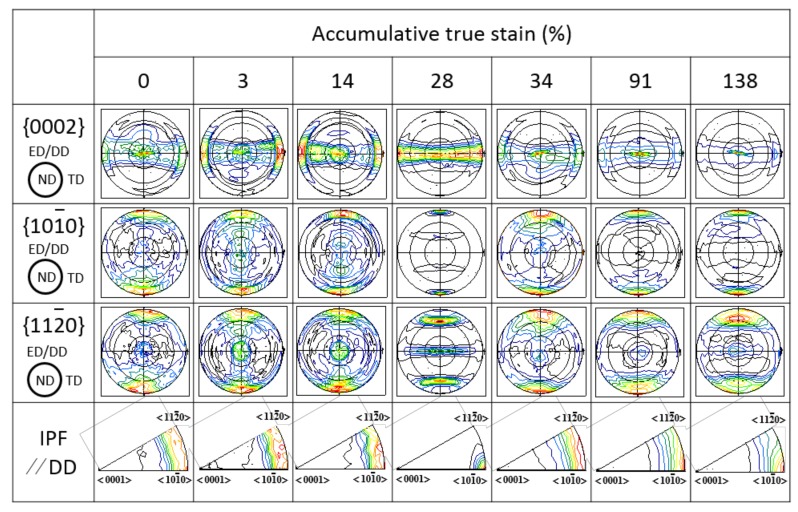
The pole figures (PF) and inverse pole figures (IPF) of as-extruded and as-drawn Mg wire with ATS of 3%, 14%, 28%, 34%, 91% and 138%.

The texture of Mg wire in the form of pole figures (PF) including {0002}, {10$\bar{1}$0}, {11$\bar{2}$0} and inverse pole figures (IPF) parallel to the DD is displayed in Figure 3. The as-extruded pure Mg exhibits a strong texture with the basal plane {0002} aligned almost parallel to the ED. With strain imposed (3~28%), the {0002} pole figures indicate that the c-axis of most grains tilt away from ND toward TD, and the new texture components appear, which are tilted about 90° or 60° from ND toward TD. Meanwhile, both {10$\bar{1}$0} and {11$\bar{2}$0} obviously rotate and generate new texture components perpendicular to and 60° to the DD, respectively. Up to ATS ~34%, the texture has changed obviously with most c-axis aligned along the ND again, which is similar to the PF of as-extruded pure Mg. With increasing ATS (~91%, ~138%), the basal texture is much more concentrated. Besides, in the IPF parallel to the DD, the texture component gradually forms the ideal <10$\bar{1}$0> fiber texture at ATS ~28%; and in the subsequent ATS ~34%, this fiber texture is not obvious. However, as the strain increase to ~138%, the weaker <100> fiber texture is gradually formed again. This texture evolution features are quite different from that of Mg2Zn which the strongest texture component gradually transforms from as-extruded basal to <100> fiber at strain ~91% [7]. So for, this special texture evolution has not been seen in other studies. This finding is expected to provide an important new concept for understanding the mechanical properties evolution and further for developing highly formable Mg products.

**Figure 4:**
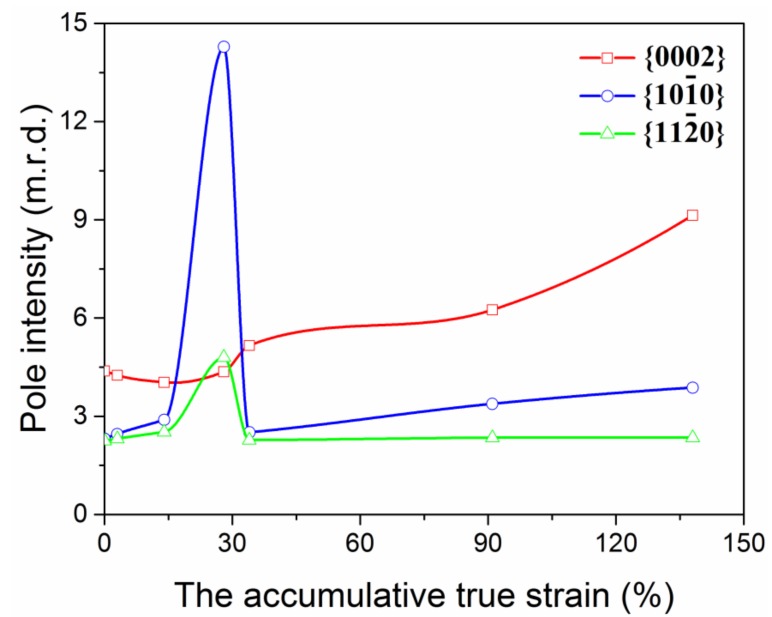
The maximum pole density of typical planes of as-drawn Mg wire with different ATS.

The maximum pole density data obtained from PF is presented in Figure 4a. A slight decrease intensity from 4.38 to 4.25 multiples of random distribution (m.r.d.) in the plane {0002} is seen at the initial stage of cold drawing. Afterwards the curve keeps a steady rising tendency, and reaches 9.14 m.r.d. at ATS ~138%. In sharp contrast to {0002} pole density, the intensity of {100} rapidly increases from 2.31 to 14.28 m.r.d. at ATS 0~28%, but subsequently a quick decline occurs until ~34% ATS. Afterwards the curve keeps a continuous rising tendency and finally reaches 3.87 m.r.d. The similar trend is also seen for {110}, but with much smaller ranges.

## 4. Discussion

During cold drawing for pure Mg, DRX occurred at room temperature along with low dislocation density. The microstructural features are distinctly different from that of cold-drawn Mg alloys accompanied by high density dislocations [7], but agree with the report on HPT processing of pure Mg by Edalati et al. [17].

**Figure 5:**
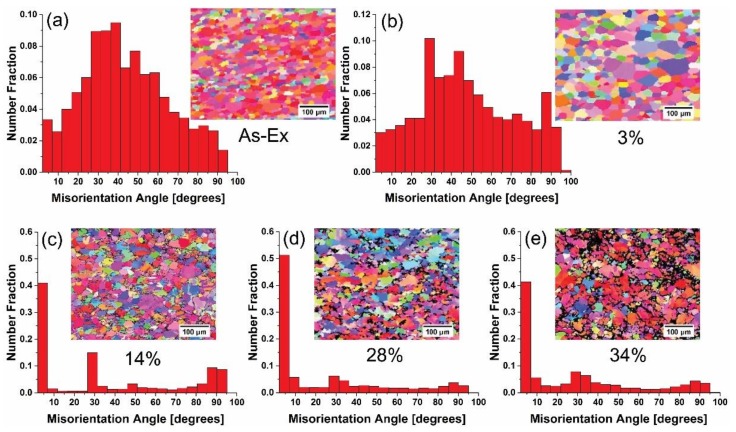
Misorientation angle distributions for pure Mg wire with different ATS: (**a**) as-extruded, (**b**) 3%, (**c**) 14%, (**d**) 28% and (**e**) 34%.

In order to gain a better understanding of the changes in the microstructure of pure Mg wire during cold drawing, the misorientation angle distribution for as-extruded and as-drawn Mg wires obtained from the EBSD maps are shown in Figure 5. It is noted that there are black areas in the maps, where the confident index value in EBSD data collection is lower than 0.3. Since the quality of Kikuchi patterns in EBSD data is low at large ATS, as-drawn Mg wires with only lower ATS were examined using EBSD and the texture of pure Mg wire with lower and higher strains was tested by XRD macrotexture in this work. From Figure 5a, it appears that as-extruded Mg wire has a misorientation angle distribution which is dominated by high angle boundaries. With increasing strain (3~28%), the rate of low angle boundaries (<10°) clearly increases (Figure 5b–d), which is result from agglomeration of dislocations, as shown in Figure 2d. Up to ~34% ATS, the fraction of high angle boundaries rises (Figure 5e). This is due to the dislocations annihilation. As the misorientation angle increases, dislocations are more likely to be absorbed at the boundaries [21]. It can be found that the misorientation angle evolution coincides with the evolution of the microstructure (Figure 2). The distinct microstructure of as-drawn pure Mg has a critical impact on the evolution of mechanical properties.

When Mg alloys are processed by cold drawing, the hardness and strength are generally rising with increasing strain, showing a significant work hardening [7,8,9,10]. By contrast, the hardness and strength of pure Mg in this study show a distinct evolution, which is divided into three stages (Figure 1).

Firstly, hardness and strength gradually increase, reaching a peak value at ATS ~28%, and thereafter there is a sharp decrease, finally saturating to steady-state levels. This work-hardening was caused mainly by the proliferation and accumulation of dislocations [22,23]. With increasing strain (~28% ATS), slip systems were gradually activated, resulting in a significant increase in dislocation density and the formation of dislocation cells, as shown in Figure 2d. As ATS continued to increase, the cross slip was activated. More dislocations were gathered in the subgrain boundary, causing dislocations to annihilate and recombine. In addition, high internal stresses also promoted the migration of grain boundaries and high angle boundaries gradually increased. The subsequent work-softening is attributed to the annihilation of dislocations in grain boundaries.

For pure Mg, due to its low melting temperature, ultrahigh purity and high stacking fault energy (SFE), the mobility of dislocation is high. The correlation research [14] indicated that when the grain size was large, the dislocations could accumulate in the interior of grains and form dislocation cells. However, when the grain size became smaller, the deformation-induced dislocations were recovered in grain boundaries due to their high mobility of dislocations.

The formation of steady-state level was a result of a balance between the hardening (the proliferation and accumulation of dislocations) and the softening (the annihilation of dislocations) owing to grain boundary migration [24] or recovery/recrystallization [25]. Based on the experimental results including microhardness-strain curve (Figure 1), TEM microstructures (Figure 2) and the grain boundary characteristics (Figure 5e), it can be concluded that DRX have occurred after large ATS drawing, effectively releasing the stored strain energy and consequently facilitating the further deformation. 

Moreover, the DRX contributes to the texture evolution which is also distinctly different from Mg2Zn alloy [7] and has not been presented in other documents. The strongest texture component gradually transformed from as-extruded basal to <100> fiber at ATS ~28%, which is similar to that of Mg2Zn at maximum ATS ~91%. In the subsequent strain (>~28%), the texture rapidly returned to the clear basal {0002} with an obviously reduction in texture intensity (Figure 4).

**Figure 6:**
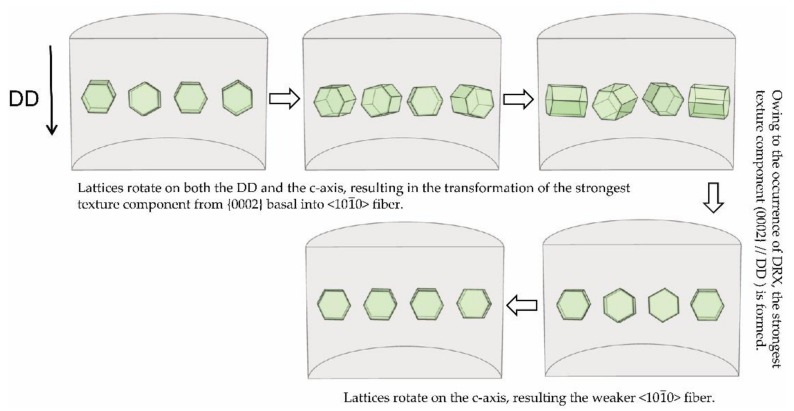
Schematic drawing of lattice rotation in the process of pure Mg cold drawing.

Based on macro-textural analysis, the rotation processing of Mg lattices during cold drawing is schematically illustrated in Figure 6. Lattice rotation is a result of cold-drawing deformation and directly reflects the deformation mode. Hence, it is necessary to further analyze the cold-drawing deformation process. As Figure 6 shows, three principal process should be noted: (i) On the one hand, lattices rotate on the DD and become more uniformly distributed around the circumference, simultaneously accompanied by tilting towards the DD, while on the other lattices rotate on c-axis and from <110>//DD to <100>//DD. Namely, the strongest texture component transfers from as-extruded basal into <100> fiber. This stage is very short, only approximately 28% ATS, but the texture intensity greatly increases, and thus the hardness and strength increase, showing a significant work hardening; (ii) Owing to the occurrence of DRX at room temperature, the basal texture component, {0002}//DD, is formed again. Moreover, owing to the different orientation between the new grain and the deformed grain, the texture intensity is weakened significantly. Therefore, the hardness and strength represent a falling trend, showing an obvious work softening; (iii) Lattices rotate on c-axis and, as the strain increases, the weaker <10$\bar{1}$0> fiber texture is gradually formed again. Only a slight rotation occurs for Mg lattices and the texture intensity slightly increases. At this stage, the hardness and strength maintain a stable level. It can be seen that different lattice rotation processes have occurred during cold drawing, corresponding to different evolution of mechanical properties.

The texture plays a very important role in Mg and its alloys for mechanical properties. Texture strengthening of pure Mg is generally caused by cold drawing along DD which limits the basal slip and is beneficial to the activation of the twins and non-basal slips, thus increasing the hardness and strength, as is revealed for Mg2Zn [7] and Mg at lower ATS (<~28%). Texture weakening for pure Mg is achieved at larger ATS due to DRX which randomizes the orientation, thus decreasing the hardness and strength. This, in turn, can greatly improve the deformability of pure Mg wires.

These differences in microstructures and mechanical properties are attributed to the different rates of recovery in pure Mg and Mg2Zn. Pure Mg has a very high SFE, the partial dislocations are not widely separated, and therefore DRX are easy through cross-slip accompanied by the re-formation of weak basal texture. The above-mentioned combined effects reduce the hardness and strength at larger ATS. In contrast, Mg2Zn has a lower SFE coupled with the solute drag effect of Zn. In Mg2Zn, the recovery occurs more slowly, and thus DRX are difficult to occur, which makes the hardness and strength rise. Direct evidence for this difference is readily available by comparing two sets of micrographs, mechanical properties and textures for Mg2Zn and pure Mg. Based on the above analysis, the distinct microstructure, texture modification introduced by cold drawing influence the mechanical properties and significantly improve the deformability of pure Mg wires.

## 5. Conclusions

To conclude, in this study, pure Mg was subjected to multiple-pass cold drawing that showed super-formability (~138% ATS) at room temperature. The hardness and strength began to rise with increasing strain. After reaching to the maximum value at ~28% ATS, the hardness and strength simultaneously decreased to a steady-state level owing to the DRX. In addition, the distinct texture evolution was also responsible for the distinct evolution in hardness and strength. The texture was mainly characterized by initially transformed from as-extruded basal to <10$\bar{1}$0> fiber (~28% ATS) accompanied by the texture intensity increasing, and then rapidly returned to the basal ({0002}//DD) with the intensity dramatically declining. The strong tendency for work softening and texture modification result in highly ductile Mg wires, which may be expected to demonstrate significantly enhanced formability.
